# Cuproptosis-related molecular subtypes direct T cell exhaustion phenotypes and therapeutic strategies for patients with lung adenocarcinoma

**DOI:** 10.3389/fphar.2023.1146468

**Published:** 2023-04-11

**Authors:** Yi-Pan Zhu, Hui-Ting Deng, Xiuyu Wang, Michal A. Rahat, Shupeng Sun, Qiang-Zhe Zhang

**Affiliations:** ^1^ State Key Laboratory of Medicinal Chemical Biology and College of Pharmacy, Tianjin Key Laboratory of Molecular Drug Research, Nankai University, Tianjin, China; ^2^ Tianjin Key Laboratory of Extracorporeal Life Support for Critical Diseases, Institute of Hepatobiliary Disease, Tianjin Third Central Hospital, Nankai University, Tianjin, China; ^3^ Department of Neurosurgery, Tianjin First Central Hospital, School of Medicine, Nankai University, Tianjin, China; ^4^ Immunotherapy Laboratory, Carmel Medical Center, and the Ruth and Bruce Rappaport Faculty of Medicine, Technion-Israel Institute of Technology, Haifa, Israel; ^5^ Department of Neurosurgery, Tianjin Huanhu Hospital, School of Medicine, Nankai University, Tianjin, China

**Keywords:** cuproptosis, T cell exhaustion, tumor immune microenvironment, lung adenocarcinoma, immunotherapy

## Abstract

**Background:** T cell exhaustion (TEX) heterogeneity leads to unfavorable immunotherapeutic responses in patients with cancer. Classification of TEX molecular phenotypes is pivotal to overcoming TEX and improving immunotherapies in the clinical setting. Cuproptosis is a novel form of programmed cell death associated with tumor progression. However, the relation between cuproptosis-related genes (CuRGs) and the different TEX phenotypes has not been investigated in lung adenocarcinoma (LUAD).

**Methods:** Unsupervised hierarchical clustering and principal component analysis (PCA) algorithm were performed to determine CuRGs-related molecular subtypes and scores for patients with LUAD. The tumor immune microenvironment (TIME) landscape in these molecular subtypes and scores was estimated using ESTIMATE and ssGSEA algorithms. Furthermore, TEX characteristics and phenotypes were evaluated in distinct molecular subtypes and scores through GSVA and Spearman correlation analysis. Finally, TIDE scores, immunophenoscore, pRRophetic, GSE78220, and IMvigor210 datasets were employed to appraise the distinguishing capacity of CuRGscore in immunotherapy and pharmacotherapy effectiveness.

**Results:** We identified three CuRGclusters, three geneClusters, and CuRGscore based on 1012 LUAD transcriptional profiles from five datasets. Compared with other molecular subtypes, CuRGcluster B, geneCluster C, and low-CuRGscore group with good prognosis presented fewer TEX characteristics, including immunosuppressive cells infiltration and TEX-associated gene signatures, signal pathways, checkpoint genes, transcription and inflammatory factors. These molecular subtypes were also responsive in distinguishing TEX phenotype in the terminal, GZMK+, and OXPHOS- TEX subtypes, but not the TCF7+ TEX subtype. Notably, copper importer and exporter, SLC31A1 and ATP7B, were remarkably associated with four TEX phenotypes and nine checkpoint genes such as *PDCD1*, *CTLA4*, *HAVCR2*, *TIGIT*, *LAG3*, *IDO1*, *SIGLEC7*, *CD274*, *PDCD1LG2*, indicating that cuproptosis was involved in the development of TEX and immunosuppressive environment in patients with LUAD. Moreover, CuRGscore was significantly related to the TIDE score, immunophenoscore, and terminal TEX score (Spearman R = 0.62, *p* < 0.001) to effectively predict immunotherapy and drug sensitivity in both training and external validation cohorts.

**Conclusion:** Our study demonstrated the extensive effect of cuproptosis on TEX. CuRGs-related molecular subtypes and scores could illuminate the heterogeneity of TEX phenotype as reliable tools in predicting prognosis and directing more effective immunotherapeutic and chemotherapeutic strategies for patients with LUAD.

## 1 Introduction

Lung cancer is the leading cause of cancer-related deaths worldwide (Sung et al., 2021), with a 5-year survival rate of only 22% (Siegel et al., 2022). Approximately 85% of lung cancer cases are non-small cell lung cancer (NSCLC), of which lung adenocarcinoma (LUAD) are the major subtypes (Molina et al., 2008; Herbst et al., 2018). The carcinogenesis of LUAD is associated with smoking, alcohol consumption, metabolic disorders, and other factors (Alberg et al., 2013), which cause molecular and pathological alterations, genetic mutations, and immune escape in tumor (Zhang et al., 2021). The death rate of LUAD is high due to lack of effective diagnostics and treatment.

Immune checkpoint blockade (ICB) therapy has revolutionized the treatment of advanced NSCLC. It has improved the overall survival of NSCLC patients, but only a few patients have benefited in the clinical setting (Jiang et al., 2021). The objective response rate to PD-1 blockade (Pembrolizumab) is 19.4% for all the patients with a median overall survival of 12.0 months (Garon et al., 2015). In another monotherapy of anti-PD-1, median overall survival of Nivolumab was 9.9 months in 129 patients with an objective response rate of 17% (Gettinger et al., 2015). Functional phenotypes of T cells are considered as key deciders for immunotherapy efficiency.

Tumor-infiltrating CD8^+^ T cells serve as primary executors that can recognize and kill tumor cells (Durgeau et al., 2018). They encounter immunosuppressive signals in the tumor microenvironment (TME) respond by becoming exhausted, and multiple subsets of T cell exhaustion (TEX) were identified (Crespo et al., 2013; Cai et al., 2020). Four TEX subsets have been defined based on Ly108 and CD69 expression: TEX progenitor 1 (Ly108+ CD69^+^) and 2 (Ly108+ CD69^−^), TEX intermediate (Ly108- CD69^−^), and TEX terminal (Ly108- CD69^+^) (Beltra et al., 2020). Progenitor and intermediate TEX selectively respond to anti-PD-1 therapy, while terminal TEX cells do not (Blackburn et al., 2008; Miller et al., 2019; Beltra et al., 2020). The heterogeneity of TEX could obstruct the favorable therapeutic response in patients with cancer (Dolina et al., 2021). Therefore, a deeper understanding and microdissection of TEX heterogeneity that would point to possible new biomarkers are crucial to alleviate TEX and improve tumor immunotherapy efficacy and clinical outcomes.

Cuproptosis is recently identified as a new copper-dependent form of regulated cell death, which is different from other known regulated cell death (Tang et al., 2022; Tsvetkov et al., 2022; Wang et al., 2022). Accumulation of intracellular copper induces mitochondrial stress through the aggregation of lipoylated tricarboxylic acid cycle enzymes and the loss of Fe–S cluster proteins, and ultimately causes cuproptosis (Chen et al., 2022; Tsvetkov et al., 2022). Copper (Cu) ionophores such as elesclomol raising intracellular Cu concentration induced cell death and executed antineoplastic activity (Tsvetkov et al., 2022; O'Day et al., 2013). Similarly, artificially overexpressing copper importer SLC31A1 resulted in Cu over-accumulation, thereby triggered cuproptosis in A549 lung cancer cells (Tsvetkov et al., 2022). Whereas, depleting cupric ion with copper chelators reduced PD-L1 expression by both inhibiting PD-L1 transcription and promoting PD-L1 ubiquitination degradation in xenograft mouse (Voli et al., 2020). The evidence suggested that cuproptosis was tightly associated with tumor progression. However, the relationship between cuproptosis-related genes (CuRGs) dissecting molecular subtypes and TEX phenotypes in patients with LUAD was largely unknown.

In the study, we chose thirteen previously identified cuproptosis-related genes (CuRGs) and four gene expression omnibus (GEO) datasets with the same platform GPL570 as candidate genes and data to hold the accuracy of model building. The principal component analysis (PCA) algorithm considers the contribution of each gene in a large gene set rather than several genes to define the CuRGscore for each patient; hence, we employed it together with unsupervised hierarchical clustering to construct three CuRGclusters, three geneClusters, and CuRGscore based on 1012 patients with LUAD in the training cohort. Two independent GEO datasets and two pre-treatment immunotherapy response datasets were used to validate the accuracy of the results. Finally, our results concluded that these molecular subtypes and CuRGscores could distinguish the heterogeneity of TEX phenotype to predict prognosis and immuno- and chemotherapeutic responses reliably in patients with LUAD.

## 2 Materials and methods

### 2.1 LUAD data sets and preprocessing

**FIGURE 1:**
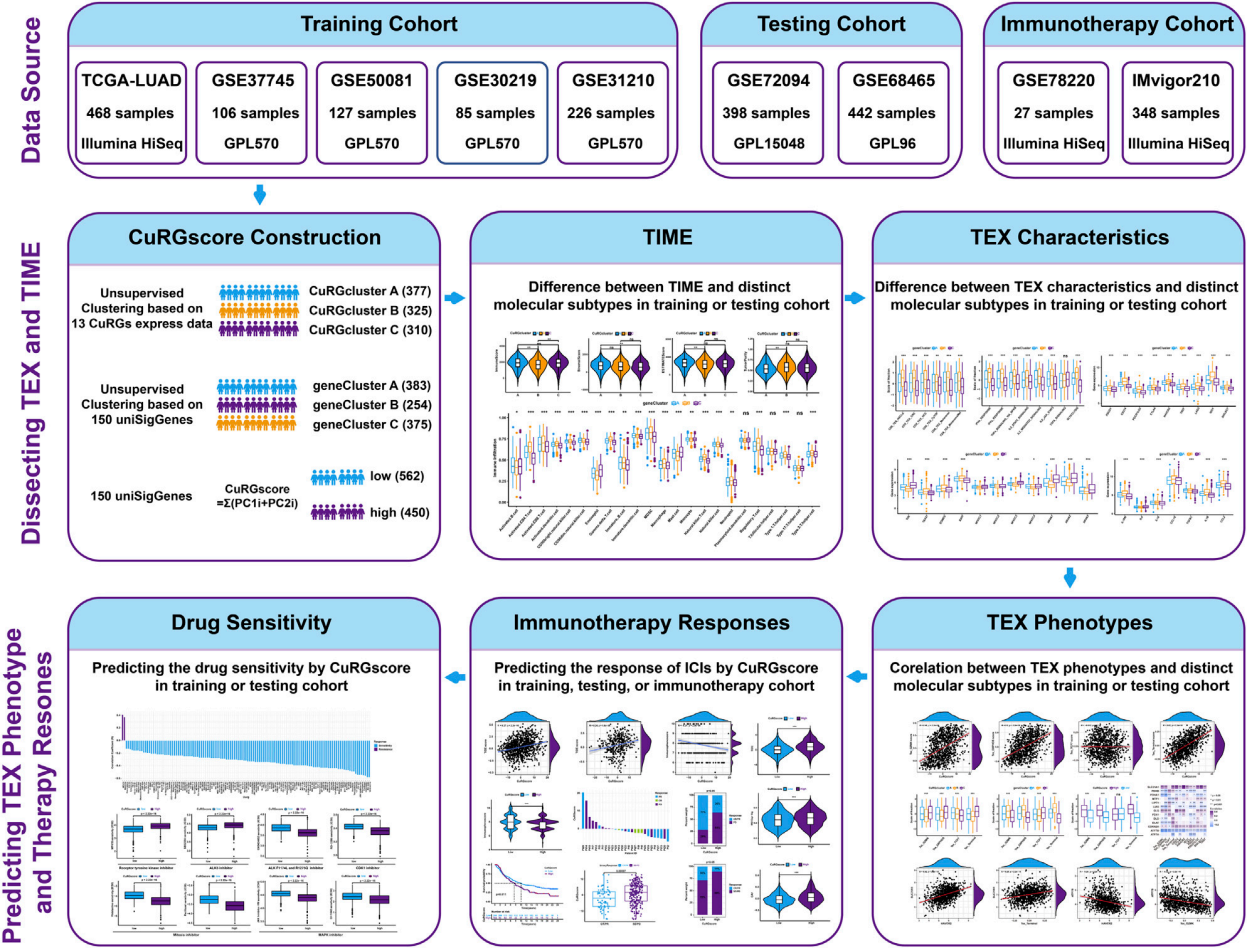
The flow diagram of the study.

The flow diagram of the study is illustrated in Figure 1. The transcriptome data and clinical information of patients with LUAD were obtained from The Cancer Genome Atlas (TCGA) repositories (https://portal.gdc.cancer.gov). Six microarray datasets, namely, GSE30219, GSE31210, GSE37745, GSE50081, GSE72094, and GSE68465, were downloaded from the GEO database (https://www.ncbi.nlm.nih.gov/geo/). Information about these datasets is described in detail in Supplementary Table S1. Four GEO datasets (GSE30219, GSE31210, GSE37745, and GSE50081 from the same platform GPL570) and the TCGA-LUAD (Illumina HiSeq) dataset were chosen as training cohort, while two independent GEO datasets (GSE72094 from the GPL96 platform and GSE68465 from the GPL15048 platform) were used as external validation cohorts. Samples without documented survival information were excluded. The fragments per kilobase million (FPKM) values in the TCGA-LUAD dataset were converted to transcripts per kilobase million (TPM) values for our analysis (Conesa et al., 2016; Gustafsson et al., 2021). For GEO datasets, the expression of genes with multiple probes was averaged. Then the “Combat” algorithm of the SVA package was used to correct the batch effect of samples and merge the training data into one dataset (Zhang et al., 2020). Based on the above procedures, 1012 patients with LUAD in the training dataset and 840 in the testing cohort were collected for subsequent analysis.

### 2.2 Unsupervised hierarchical clustering for CuRGclusters and geneClusters

Firstly, 13 reported genes were chosen as CuRGs in the study, including 3 genes of copper transporter protein (CuPT; *ATP7A*, *ATP7B*, and *SLC31A1*), 4 components in lipoic acid (LA) pathway (*FDX1*, *LIAS*, *LIPT1*, and *DLD*), 3 pyruvate dehydrogenase (PDH) complex (*DLAT*, *PDHA1,* and *PDHB*) and other three proteins (*MTF1*, *GLS,* and *CDKN2A*) (Tsvetkov et al., 2022). Based on the expression level of the 13 CuRGs, 1012 patients with LUAD were categorized into distinct molecular subtypes using the “ConsensusClusterPlus” R package. To ensure cluster stability, 1,000 iterations were performed (Wilkerson and Hayes, 2010). The clusters were identified according to the optimal k value of the consensus matrix in the training cohort and were named CuRGclusters. Subsequently, gene expression profiles were analyzed between these CuRGclusters using an empirical Bayesian approach in the “limma” R package (Ritchie et al., 2015). The differentially expressed genes (DEGs) from different CuRGclusters with adjusted *p*-values <0.001 were considered statistically significant. The univariate Cox regression analysis was used to process the DEGs to extract the expression profile of significant prognostic genes. Based on these prognostic DEGs, 1012 patients with LUAD were dissected again by unsupervised hierarchical clustering. The clusters were identified according to the optimal k value and were termed geneClusters.

### 2.3 Construction of the CuRGscore

CuRGs-related gene signature was developed using the PCA algorithm based on the prognostic-related DEGs, named CuRGscore. Principal components 1 and 2 were extracted to serve as the signature score using a method similar to GGI (Sotiriou et al., 2006; Zhang et al., 2020). The CuRGscore for each patient was calculated using: CuRGscore = Σ(PC1_i_ + PC2_i_), where is the expression of these significant prognostic genes. The patients were divided into high- and low-CuRGscore groups according to the optimal cutoff value derived from the Survminer algorithm. Kaplan-Meier survival analysis was executed to evaluate the overall survival (OS) of patients with LUAD (log-rank test, *p* < 0.001).

### 2.4 Evaluation of TEX molecular characterization in different molecular subtypes

Six gene signatures of CD8 TEX were chosen from previous studies to investigate the molecular characterization of TEX in different molecular subtypes, including NSCLC (Guo et al., 2018), colorectal cancer (Zhang et al., 2018), hepatocellular carcinoma (Zheng et al., 2017), melanoma (Sade-Feldman et al., 2018), metastatic melanoma (Tirosh et al., 2016) and chronic infection (Bengsch et al., 2018). Four gene signatures of the TEX phenotype were collected from pan-cancer single-cell sequencing data (Zheng et al., 2021). Eight signaling pathways and hallmark genesets associated with TEX were extracted from the Molecular Signatures Database (MSigDB, V7.5.1) (Liberzon et al., 2015). These TEX signatures were further used to conduct gene set variation analysis (GSVA) to evaluate the TEX stage in CuRGclusters, geneClusters, and CuRGscore. Subsequently, checkpoint genes, transcription, and inflammatory factors associated with TEX were further analyzed in different molecular subtypes. Nine checkpoint genes such as *PDCD1*, *CTLA4*, *HAVCR2*, *TIGIT*, *LAG3*, *IDO1*, *SIGLEC7*, *CD274*, *PDCD1LG2* were chosen as TEX-associated checkpoint genes (Yang et al., 2022). Furthermore, eleven transcription factors, including *TOX*, *TBX21*, *EOMES*, *BATF*, *NFATC1-4*, *NR4A1*-3, as well as seven inflammatory factors such as *IL1RN*, *IL4*, *IL10*, *CCL18*, *TGFB1*, *IL1B*, *CCL2* were employed to evaluate the TEX progress (Zheng et al., 2021; Zhang et al., 2022). Finally, the difference in these genes was compared between distinct subtypes using the “limma” R package.

### 2.5 Correlation analysis for CuRGscore and TEX characterization

To uncover the correlation between CuRGscore and TEX progress, Spearman correlation analysis was conducted between CuRGscore and four TEX phenotypes. A *p*-value less than 0.05 was considered statistically significant. Moreover, CuRGs were extracted and normalized by the limma R package. The correlation of CuRGs with TEX phenotypes and checkpoint genes was estimated with the Spearman analysis.

### 2.6 Stratification analyses between CuRGscore and TEX phenotype or immune checkpoint genes

Patients were divided into subgroups according to the four TEX phenotypes or the nine immune checkpoint genes. Subsequently, the Kaplan-Meier survival curve of patients in each subgroup was analyzed to evaluate the stability of CuRGscore prognostic efficacy (log-rank test, *p* < 0.01).

### 2.7 Evaluation of TIME in different molecular subtypes

The cell infiltration of LUAD samples with the immune, stromal, and ESTIMATE scores, as well as tumor purity, were evaluated using the ESTIMATE R package (Yoshihara et al., 2013). A higher stromal score or immune score indicates a higher relative content of stromal or immune cells in the TME, while an ESTIMATE score indicates an aggregated TME stromal or immune score. Tumor purity represents the amount of tumor cells in TME. Subsequently, the ssGSEA algorithm was used to assess the abundance of 23 infiltrated immune cells in TIME for each LUAD sample (Barbie et al., 2009).

### 2.8 Assessment of immunotherapeutic response of CuRGscore

To test the predictive ability of CuRGscore in immunotherapy, the immunophenoscore was downloaded from the cancer immunome atlas (TCIA, https://tcia.at) (Charoentong et al., 2017). Tumor immune dysfunction and exclusion (TIDE) score, another robust predictor for immunotherapy, was estimated by uploading the data normalized to the average to the TIDE web (http://tide.dfci.harvard.edu/) (Jiang et al., 2018; Fu et al., 2020). High immunophenoscore represents better responses to immune checkpoint blockade (ICB) therapy, while a high TIDE score implies more immune evasion. Then, the Spearman correlation was calculated between CuRGscore and immunophenoscore or TIDE score. The distribution of TIDE score or immunophenoscore was also analyzed in the high- and low-CuRGscore groups.

Moreover, the GSE78220 dataset with pre-treatment melanomas undergoing anti-PD-1 therapy and the IMvigor210 dataset with pre-treatment urothelial cancer samples underlying anti-PD-L1 treatment were employed to evaluate the predictive performance of CuRGscore in immunotherapeutic response. GSE78220 (Illumina HiSeq 2000) was downloaded from the GEO database and normalized using the limma package. IMvigor210 dataset (Illumina HiSeq 2500) was obtained from http://research-pub.gene.com/IMvigor210CoreBiologies/. The raw data were normalized by DEseq2 and converted to TPM value to calculate the CuRGscore. The distribution of distinct anti-PD-L1 clinical responses in the CuRGscore subgroups was analyzed with chi-square or Wilcoxon test.

### 2.9 Assessment of chemotherapeutic response of CuRGscore

To predict the therapeutic performance in the high- and low-CuRGscore patients with LUAD, the R package “pRRophetic” was employed to determine the half maximal inhibitory concentration (IC50) in different CuRGscore groups by ridge regression. The cutoff criteria for the spearman correlation and drug sensitivity were *p*-value <0.001, and for the |Spearman Cor| was >0.1.

### 2.10 Statistical analysis

Statistical analyses of data were performed in the R software (version 4.1.3). For comparisons between two groups, unpaired Student’s t-tests were used to analyze variables with a normal distribution, while Wilcoxon tests were used for non-normal distribution. One-way ANOVA and Kruskal–Wallis tests were employed to compare the differences between three or more groups (Hazra and Gogtay, 2016). Spearman correlation analysis then estimated the correlation coefficients. Log-rank tests were used to determine the significance of Kaplan-Meier analysis-generated OS curves. Unless noted otherwise, statistical significance in two-tailed tests was considered when *p* < 0.05.

## 3 Results

### 3.1 TEX characterization of CuRGs-related molecular subtypes in LUAD

**FIGURE 2:**
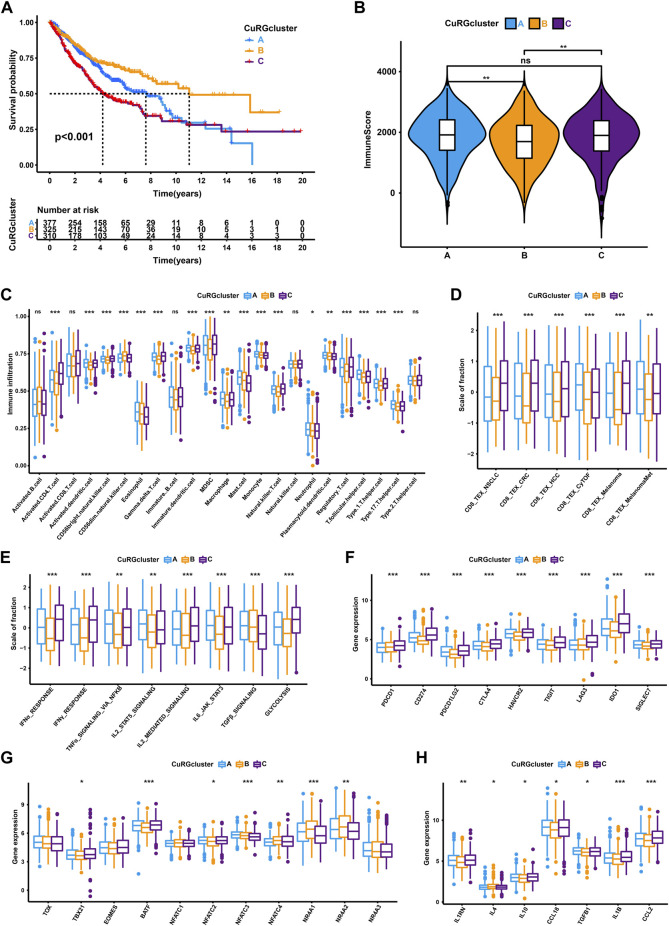
TEX and TIME characteristics of each CuRGcluster. **(A)** Survival analyses for the three CuRGclusters based on 1012 patients from the training cohort (GSE30219, GSE31210, GSE37745, GSE50081, TCGA-LUAD). **(B)** Immune score analyses using the ESTIMATE algorithm in three CuRGclusters. **(C)** The abundance of each tumor microenvironment infiltrating cell in CuRGcluster A-C. **(D–H)** GSVA enrichment analysis between CuRGcluster A-C with TEX-related signatures **(D)**, signal pathways **(E)**, immune checkpoint genes **(F)**, transcription factors **(G)**, or inflammatory factors **(H)**. The line in the box represents the median value, and the asterisk represents the *p*-value. *p < 0.05; **p < 0.01; ***p < 0.001.

Three distinct molecular subtypes were identified according to the optimal consensus matrix k = 3, which included 377 patients in cluster A, 325 in cluster B, and 310 in cluster C (Supplementary Figure S1A and Supplementary Table S2). The survival curve revealed that patients in CuRGcluster B had a prominent survival advantage, while patients in CuRGcluster C exerted an unfavorable prognosis (log-rank test, *p* < 0.001; Figure 2A). The ESTIMATE analysis showed that CuRGcluster C and A had higher immune scores than CuRGcluster B (Figure 2B). Further, we investigated the proportions of 23 types of infiltrating immune cells using ssGSEA. Consistent with ESTIMATE results, most innate and adaptive immune cells were high in CuRGcluster C, while these cells were infiltrated lowly in CuRGcluster B (Figure 2C). According to the above findings, we speculated that TEX was prevalent in high levels of immune cell infiltration CuRGcluster C with poor prognosis. Hence, TEX-associated signatures and signaling pathways were analyzed to verify this hypothesis. Six TEX signatures derived from published articles were found to be highly expressed in CuRGcluster C compared with CuRGcluster B (Figure 2D). The signaling pathways associated with TEX in CuRGcluster C, including IFNα, IFNγ, TNFα, IL2, IL6, and glycolysis, except TGFβ, were also significantly upregulated compared to those in CuRGcluster B (Figure 2E). The canonical immune checkpoint genes of TEX, such as *PDCD1*, *CD274*, *PDCD1LG2*, *CTLA4*, *HAVCR2*, *TIGIT*, *LAG3*, *IDO1,* and *SIGLEC7* were highly expressed in CuRGcluster C (Figure 2F). We further investigated the inflammatory and transcription factors involved in TEX development in the three CuRGclusters. While *TBX21*, *BATF,* and *NFATC2* were significantly upregulated, *NFATC3*, *NFATC4*, *NR4A1*, and *NR4A2* were significantly downregulated in CuRGcluster C (Figure 2G). Meanwhile, inflammatory factors, including *IL1RN*, *IL10*, *CCL18*, *TGFB1*, *IL1B* and *CCL2*, were also highly expressed in CuRGcluster C (Figure 2H). The collective data demonstrated that patients in CuRGcluster C with high TEX levels result in poor OS. In contrast, CuRGcluster B patients presented a low TEX level with a better prognosis.

### 3.2 Identification and TEX characterization of CuRGs-related gene subtypes in LUAD

**FIGURE 3:**
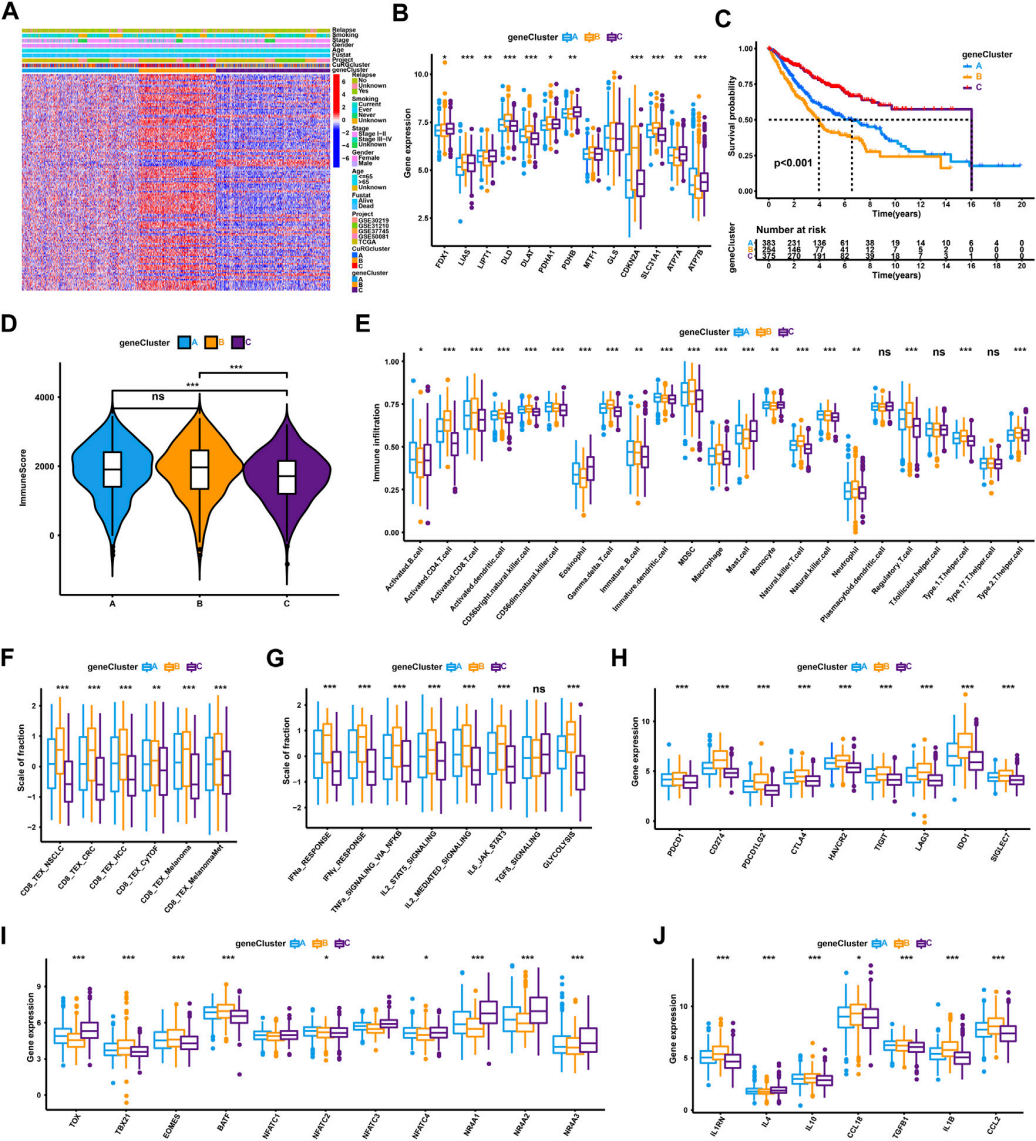
TEX and TIME characterization of the three geneClusters in LUAD. **(A)** Unsupervised clustering for 150 prognostic-related DEGs identified by univariate Cox regression classifying patients of training cohort into geneClusters A-C, respectively. The geneClusters, CuRGclusters, projects, tumor stage, survival status, relapse, smoking, gender, and age were used as clinical characteristics. **(B)** Expression of 13 cuproptosis genes in the three geneClusters. **(C)** Survival analyses for the three geneClusters based on 1,012 patients. **(D)** Immune score analyses using the ESTIMATE algorithm in the three geneClusters. **(E)** The abundance of each TME infiltrating cell in the three geneClusters. **(F–J)** GSVA enrichment analysis between geneClusters A-C with TEX-related signatures **(F)**, signal pathways **(G)**, checkpoint genes **(H)**, transcription factors **(I)**, or inflammatory factors **(J)**. The line in the box represents the median value, and the asterisk represents the *p*-value. *p < 0.05; **p < 0.01; ***p < 0.001.

Based on the optimal consensus matrix k = 3 from the unsupervised clustering of 150 prognostic-related DEGs identified by univariate Cox regression, the whole training cohort was classified into three gene clusters, defined as CuRGs-related gene clusters A-C, respectively (Supplementary Figure S1B and Supplementary Table S3). The relationship between CuRGs-related gene cluster A-C and the clinical characteristics were presented in Figure 3A. The expression of CuRGs was notably different in the three gene clusters, similar to the results with CuRGcluster A-C (Figure 3B). These results demonstrated the existence of three distinct CuRGs-classified patterns in LUAD. The Kaplan-Meier survival analysis suggested that 375 patients of gene cluster C presented the best prognosis, while 254 patients of gene cluster B appeared to have the worst OS (log-rank test, *p* < 0.001; Figure 3C). Consistent with the ESTIMATE results of CuRGclusters, geneCluster C showed low immune scores compared to geneCluster B and A (Figure 3D). Moreover, the ssGSEA results of infiltrating immune cells reconfirmed the immune score in gene cluster C, which had a low level in most innate and adaptive immune cells (Figure 3E). Together, we found that geneCluster B shows a similar result to that of CuRGcluster C, with the worst prognosis in highly immune-infiltrated subtypes in patients with LUAD. Further, geneCluster B was significantly enriched in infiltrating immune cells associated with TEX, including regulatory T cells (Treg), myeloid-derived suppressor cells (MDSC), and tumor-associated macrophages (TAM) (Figure 3E). All TEX-related signatures, signaling pathways (except TGFβ), and checkpoint genes were highly expressed in CuRGcluster B (Figures 3F–H). *TBX21*, *EOMES,* and *BATF* were considerably upregulated, whereas *TOX*, *NFAT,* and *NR4A* were remarkably downregulated in CuRGcluster B (Figure 3I). Besides IL4, other inflammatory factors were highly expressed in CuRGcluster B compared with CuRGcluster C (Figure 3J). Collectively, these results indicated that CuRGs-related geneCluster could potentially direct T cell exhaustion class to predict the prognosis of patients with LUAD.

### 3.3 Construction and TEX characterization of the CuRGscore in LUAD

**FIGURE 4:**
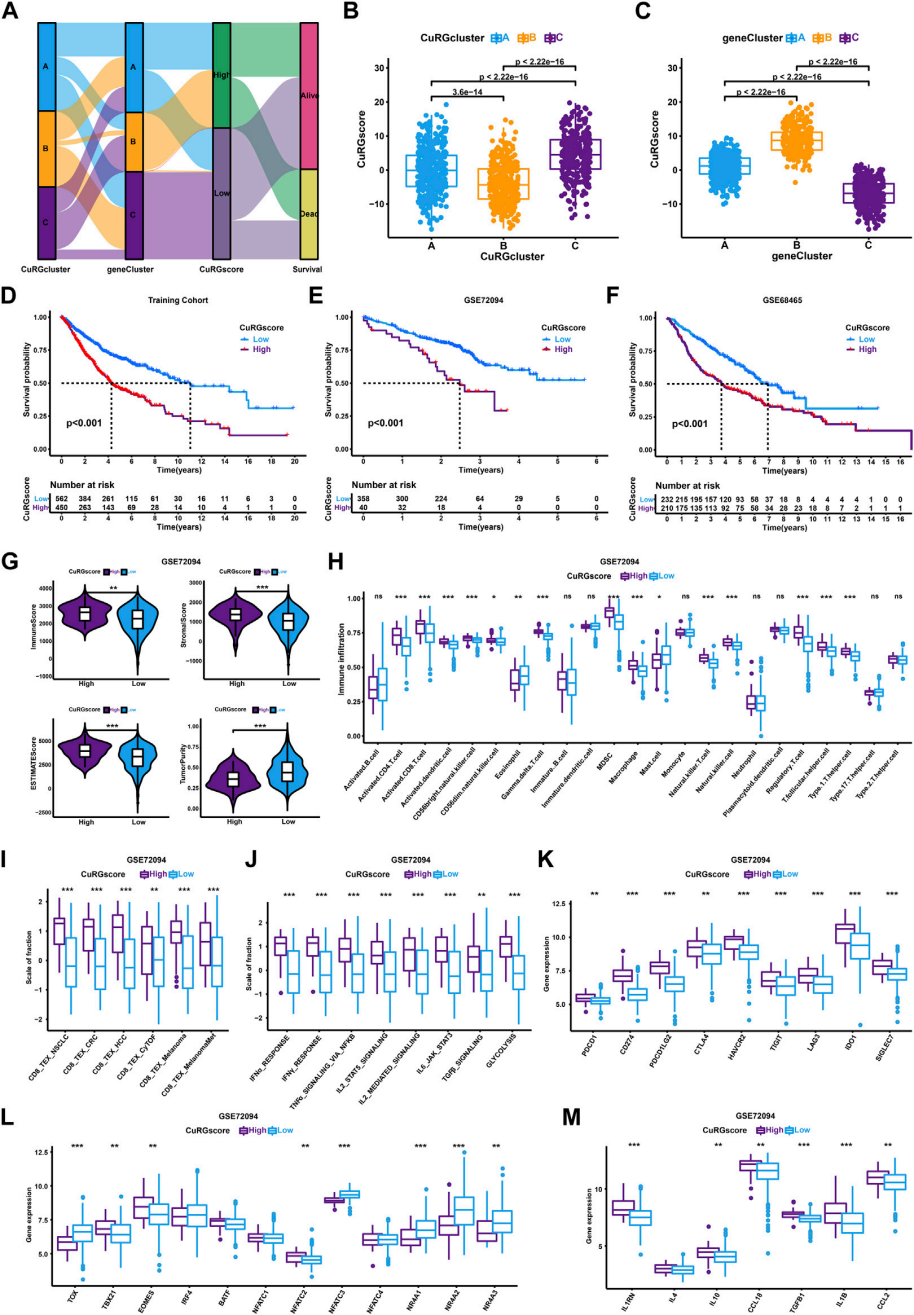
TEX and TIME Characterisation of the CuRGscore in training and external validation cohorts. **(A)** Alluvial diagram showing the relation of patients in CuRGcluster, geneCluster, CuRGscore, and survival status. **(B, C)** Differences of CuRGscore among three CuRGclusters **(B)** and geneClusters **(C)** in the training cohort (GSE30219, GSE31210, GSE37745, GSE50081, TCGA-LUAD; Kruskal–Wallis test). **(D–F)** Survival analyses for the high- and low-CuRGscore groups in the training cohort **(D)** and two independent external cohorts, GSE72094 **(E)** and GSE68465 **(F)**. **(G)** Immune, stromal, ESTIMATE, and tumor purity scores in high- and low-CuRGscore groups. **(H)** The abundance of each TME infiltrating cells in high- and low-CuRGscore groups. **(I–M) **GSVA enrichment analysis in high- and low-CuRGscore groups with TEX-related signatures **(I)**, signaling pathways **(J)**, checkpoint genes **(K)**, transcription factors **(L)**, and inflammatory factors **(M)**. The line in the box represents the median value, and the asterisk represents the *p*-value. *p < 0.05; **p < 0.01; ***p < 0.001.

To accurately quantify the grade of LUAD in individual patient, we performed a PCA algorithm to compute the CuRGscore of each tumor. Based on the cutoff value (1.034) calculated by the survminer algorithm, 1012 patients in the training cohort were divided into low- or high-CuRGscore groups (low = 562 and high = 450; Supplementary Table S4). The alluvial diagram illustrated the attribute changes of patients in CuRGclusters, geneClusters, CuRGscore, and survival status (Figure 4A). CuRGcluster B had the lowest median score, whereas the median score of subtype C was the highest (Figure 4B). On the contrary, geneCluster B also had the highest median score, while the median score of geneCluster C was the lowest (Figure 4C). Kaplan-Meier survival curves indicated that low CuRGscore signifies a better prognosis in patients with LUAD (log-rank test, *p* < 0.001; Figure 4D). This conclusion was confirmed in the external validation cohorts, including GSE72094, GSE68465, and GSE72094 + GSE68465 datasets (log-rank test, *p* < 0.001; Figures 4E, F, and Supplementary Figure S2A). We observed high immune, stromal, and ESTIMATE scores and low tumor purity in the high-CuRGscore group compared with the low-CuRGscore group (Figure 4G and Supplementary Figure S2B). Further, the high-CuRGscore group exhibited abundant TEX-related infiltrating immune cells in both training and external validation cohorts (Figure 4H and Supplementary Figure S2C). Moreover, all gene signatures, signaling pathways, and checkpoint genes of TEX were significantly increased in the high-CuRGscore group (Figures 4I–K and Supplementary Figures S2D–F). Notably, *TBX21*, *EOMES,* and *NFATC2* were significantly upregulated, while *TOX*, *NFATC3,* and *NR4A* were downregulated in the high-CuRGscore group (Figure 4L and Supplementary Figure S2G). Except for IL4, other inflammatory factors were highly expressed in the high-CuRGscore group compared with the low-CuRGscore group (Figure 4M and Supplementary Figure S2H). These findings indicated that CuRGscore could evaluate TEX phenotype and clinical outcomes of patients with LUAD in both training and external validation cohorts.

### 3.4 CuRGscore was positively associated with TEX in LUAD

**FIGURE 5:**
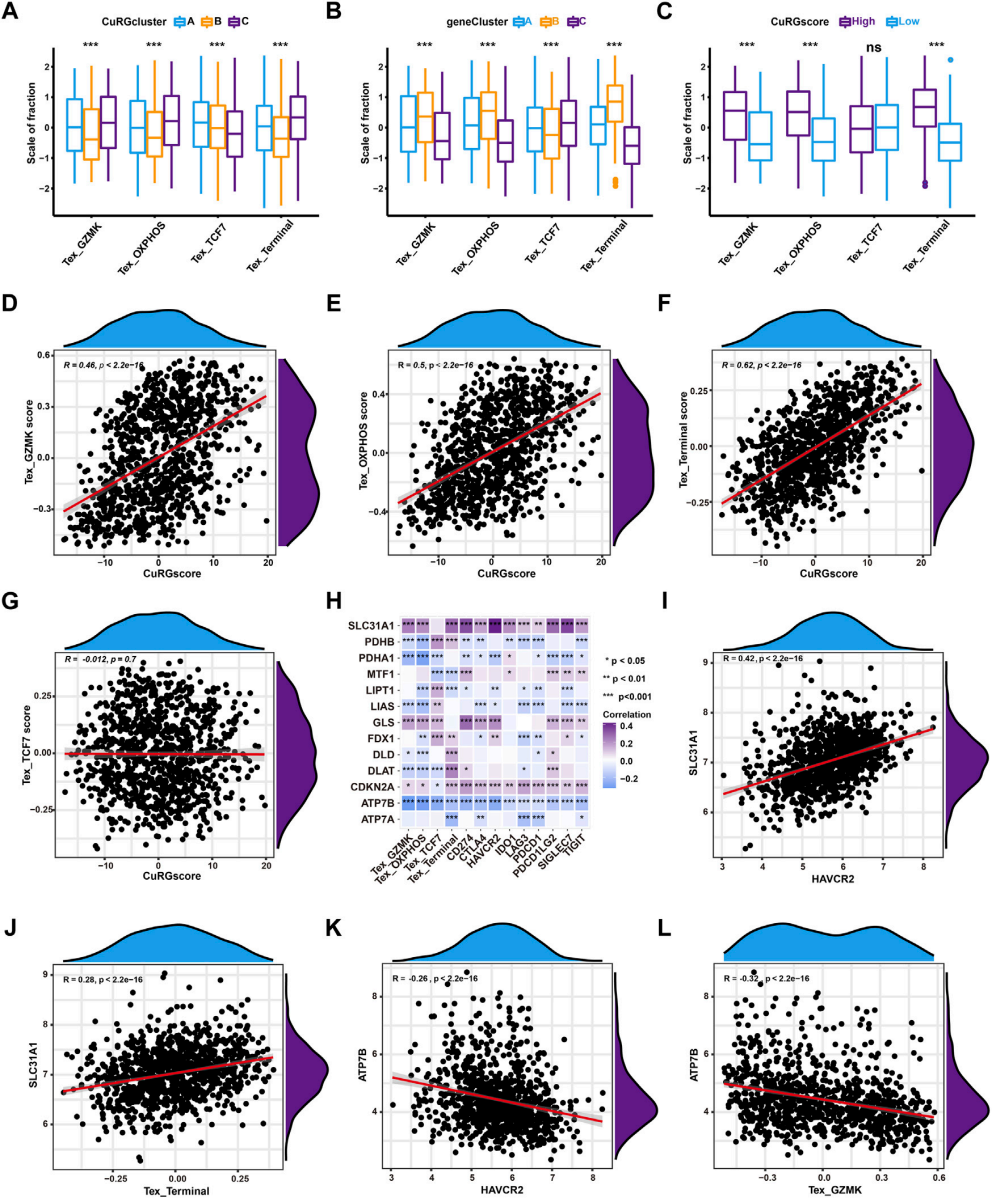
Correlation analyses between CuRGscore and TEX in the training cohort. **(A, B)** Differences in the four TEX phenotype scores among CuRGclusters A-C **(A)** or geneClusters A-C **(B)**. **(C)** Differences in the four TEX phenotype scores between high- and low-CuRGscores. **(D–G)** Spearman correlation analysis between CuRGscores and GZMK+ TEX **(D)**, OXPHOS- TEX **(E)**, Terminal TEX **(F)**, or TCF7+ TEX **(G)** scores. **(H)** Spearman correlation analyses between CuRGs and TEX markers, including TEX scores of the four subtypes and nine checkpoint genes. **(I)** Spearman correlation analysis between *HAVCR2* and *SLC31A1*. **(J)** Spearman correlation analysis between *SLC31A1* and terminal TEX score. **(K)** Spearman correlation analysis between *HAVCR2* and *ATP7B*. **(L)** Spearman correlation analysis between *ATP7B* and GZMK+ TEX score.

To further illustrate the correlation between TEX phenotypes and molecular subtypes, TEX scores were calculated for pan-cancer gene signatures of four TEX phenotypes (TCF7+, GZMK+, and OXPHOS- and terminal TEX) (Zheng et al., 2021) for 1012 patients with LUAD. Kruskal–Wallis test showed that terminal, GZMK+, and OXPHOS- TEX were significantly higher in CuRGcluster C and geneCluster B, while TCF7+ TEX was the lowest (Figures 5A, B). Similarly, terminal, GZMK+, and OXPHOS- TEX were higher in the high-CuRGscore group compared to the low-CuRGscore group (Figure 5C). Spearman correlation analyses revealed that CuRGscore was positively associated with the terminal, GZMK+, and OXPHOS- TEX scores, but not with the TCF7+ TEX score. The correlation coefficient between the terminal TEX score and CuRGscore was 0.62 (*p* < 0.001; Figures 5D–G). These results demonstrated that CuRGscore was tightly related to the terminal, GZMK+, and OXPHOS-TEX cell phenotypes. To uncover the hub genes in the terminal, GZMK+, and OXPHOS- TEX cell phenotypes caused by CuRGs, we further performed Spearman correlation analyses between the CuRGs and four TEX phenotypes or nine checkpoint genes (Figure 5H and Supplementary Table S5). We observed a significant positive correlation between *SLC31A1*, a copper importer, and the nine checkpoint genes, *PDCD1*, *CTLA4*, *HAVCR2*, *TIGIT*, *LAG3*, *IDO1*, *SIGLEC7*, *CD274*, *PDCD1LG2*. Terminal, GZMK+, and OXPHOS- TEX scores were also positively associated with *SLC31A1*. *HAVCR2* and terminal TEX scores had the strongest positive correlation with *SLC31A1* among the nine checkpoint genes and four TEX scores, respectively (Figures 5I, J). Another copper exporter, *ATP7B*, showed a significant negative association with these TEX markers. *HAVCR2* and GZMK+ TEX scores had the highest negative correlation with *ATP7B* among the nine checkpoint genes and four TEX scores, respectively (Figures 5K, L). Similar results also were found in the external validation cohort (Supplementary Figure S3 and Supplementary Table S6). Hence, the data indicated that cuproptosis, especially *SLC31A1* and *ATP7B* as copper ion importers and exporters, play an important role in the development of TEX in patients with LUAD.

### 3.5 Impact of CuRGscores, TEX phenotypes, and immune checkpoint genes on clinical outcome in LUAD

**FIGURE 6:**
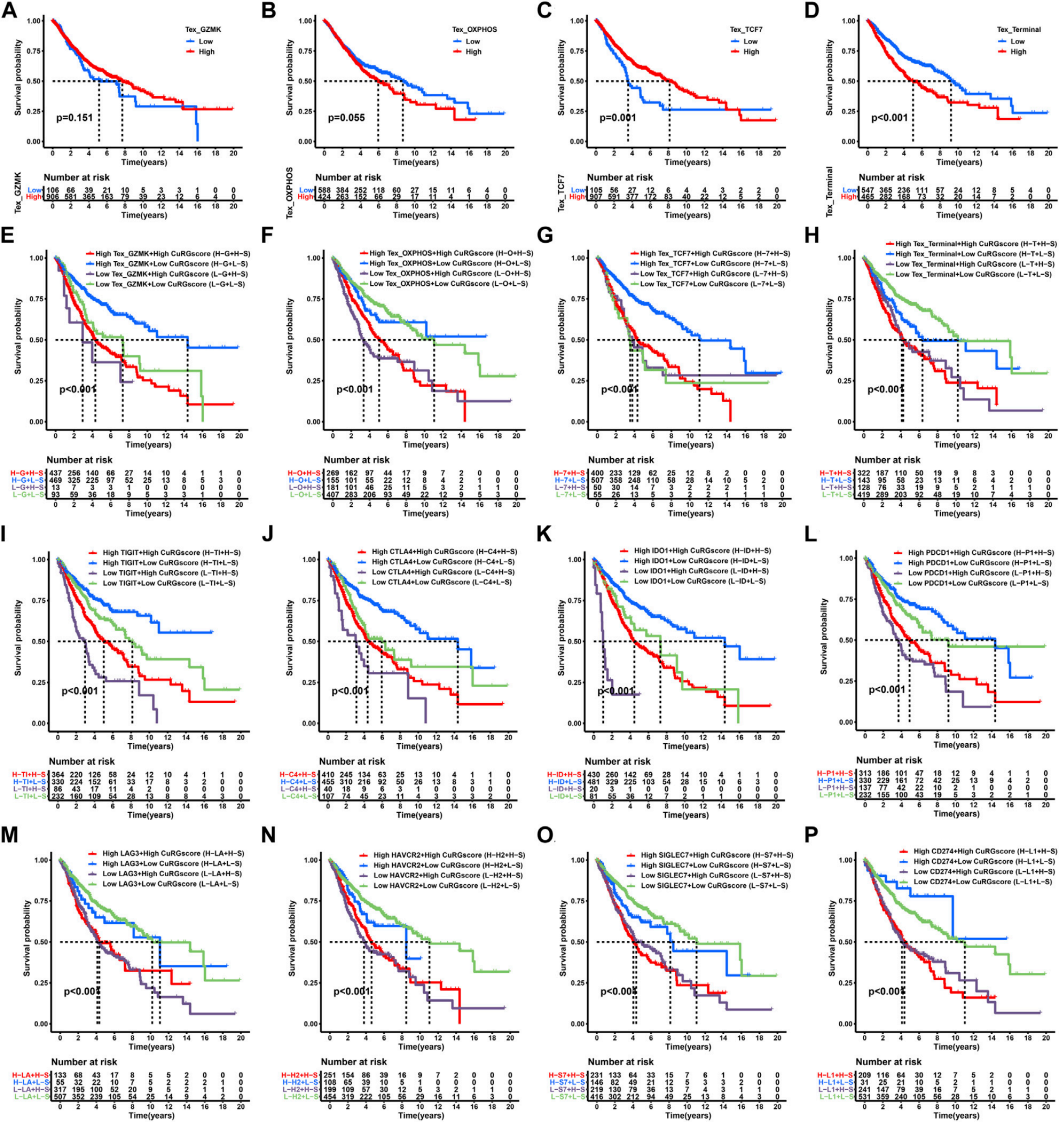
Survival analyses for subgroups of patients stratified by CuRGscores and immune checkpoint genes in the training cohort. **(A–D)** Survival analysis to examine the OS of patients with LUAD in four TEX subtypes, including GZMK+ **(A)**, OXPHOS- **(B)**, TCF7+ **(C)**, and terminal TEX phenotypes **(D)**. **(E–H)** Prognosis analysis for patients with LUAD patients stratified into four groups by CuRGscore and four TEX subtypes, including GZMK+ **(E)**, OXPHOS- **(F)**, TCF7+ **(G)**, and terminal TEX phenotypes **(H)**. **(I–P)** Kaplan-Meier curves analyzed the overall survival of patients with LUAD, stratified into four groups by CuRGscores and checkpoint genes, such as *TIGIT* **(I)**, *CTLA4* **(J)**, *IDO1* **(K)**, *LAG3* **(L)**, *PDCD1* **(M)**, *HAVCR2* **(N)**, *SIGLEC7* **(O)** and *CD274* **(P)**.

We performed a Kaplan-Meier survival analysis to examine the effect of TEX phenotypes on the OS of patients with LUAD. We found that the two intermediate phenotypes, GZMK+ and OXPHOS- TEX subtypes, do not exhibit significantly different OS between the high- and low-score groups (log-rank test, *p* > 0.05; Figures 6A, B). The early phenotype, TCF7+ TEX subtype, showed a favorable prognosis in the high-score group (log-rank test, *p* = 0.001; Figure 6C), whereas the terminal TEX phenotype demonstrated poor prognosis in the high-score group (log-rank test, *p* < 0.001; Figure 6D). We further determined whether the CuRGscores could influence the prognosis of patients with LUAD at the same TEX phenotype or similar expression level of immune checkpoint genes. The low-CuRGscore implied a significantly longer OS than the high-CuRGscore, regardless of the TEX phenotypes (log-rank test, *p* < 0.001; Figures 6E, F, H). Notably, low-CuRGscore indicated a prolonged OS of patients with a high-TCF7+ TEX score than those with a low-TCF7+ TEX score (Figure 6G). Low-CuRGscores were also predictive of a good prognosis for the TEX-related immune checkpoint genes compared with high-CuRGscores, regardless of the expression of *TIGIT* (log-rank test, *p* < 0.001; Figure 6I). Similar patterns of clinical outcomes were also found among the four groups divided by CuRGscore and *CTLA4*, *IDO1*, *PDCD1*, *LAG3*, *HAVCR2*, *SIGLEC7*, *CD274*, or *PDCD1LG2* in the training cohort (log-rank test, *p* < 0.001; Figures 6J–P and Supplementary Figure S4J). We observed similar results in the external validation cohort (GSE72094), that low-CuRGscores indicated better prognosis in patients with LUAD, regardless of the expression level of immune checkpoint genes (log-rank test, *p* < 0.01; Supplementary Figures S4A–I). These findings further confirmed that CuRGscore was a reliable biological signature for predicting the OS of patients with LUAD.

### 3.6 CuRGscore is a reliable predictor of immunotherapeutic responses in patients with LUAD

**FIGURE 7:**
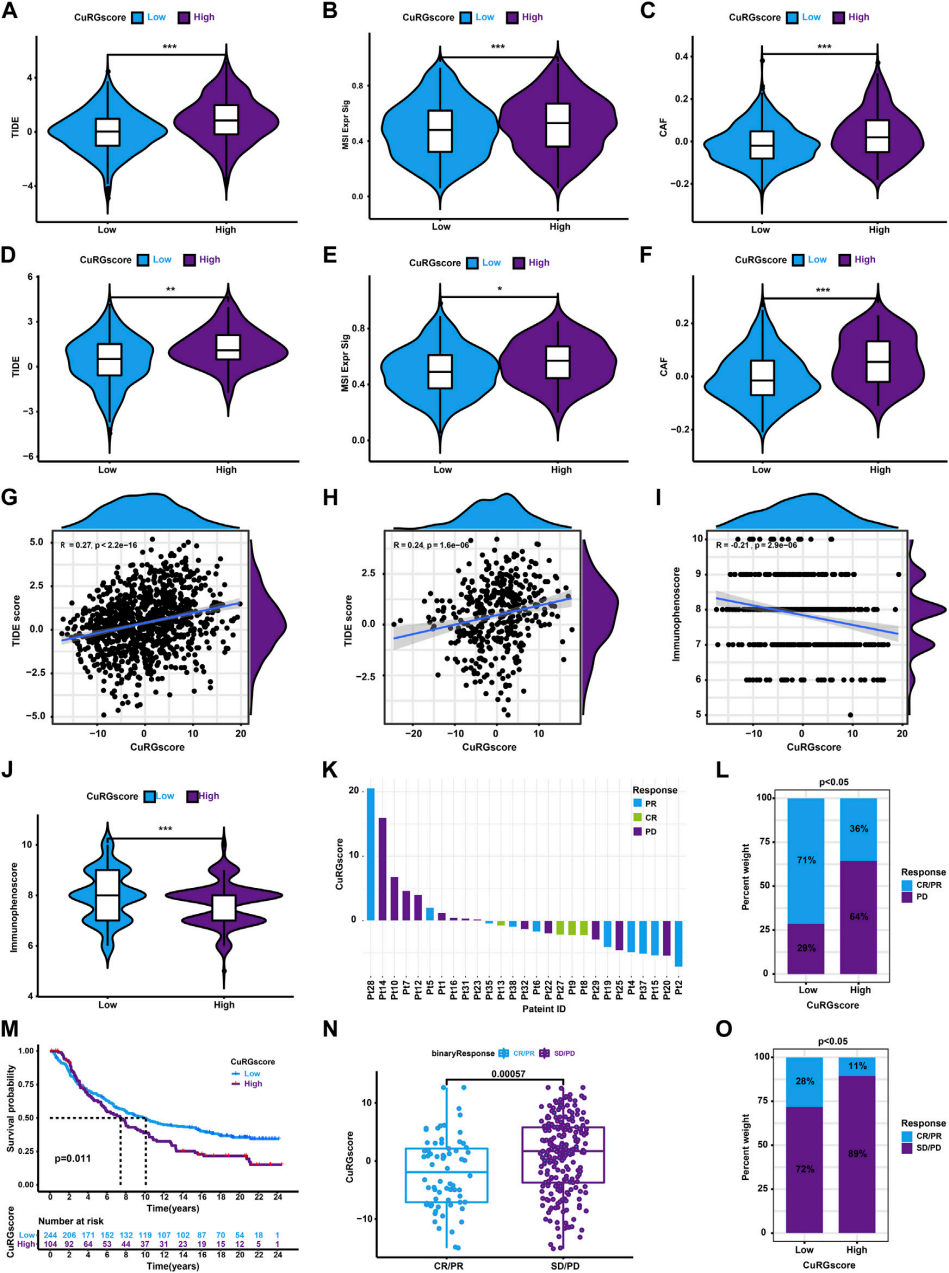
The role of CURGscore in ICB immunotherapy. **(A–C)** The distribution of TIDE **(A)**, MSI **(B)**, and CAF **(C)** scores between patients in the high- and low-CuRGscore groups in the training cohort. **(D–F)** The distribution of TIDE **(D)**, MSI **(E)**, and CAF **(F)** scores between patients in the high- and low-CuRGscore groups in the testing cohort. **(G–I)** Spearman correlation analysis for TIDE scores or immunophenoscore with CuRGscore in the training **(G)**, external testing **(H)**, and TGCA-LUAD **(I)** cohorts. **(J)** Difference of immunophenoscore between patients in the high- and low-CuRGscore groups in the TGCA-LUAD cohort. **(K)** Distribution of patients with a distinct anti-PD-1 clinical response based on CuRGscores in the GSE78220 dataset. **(L)** Distribution of distinct anti-PD-1 clinical response in CuRGscore subgroups in the GSE78220 dataset (chi-square test). **(M)** Kaplan-Meier plot of overall survival for patients of CuRGscore subgroups in the IMvigor210 cohort (log-rank test). **(N)** The difference in the CuRGscores in anti-PD-L1 clinical response subgroups in the IMvigor210 cohort (Wilcoxon test). **(O)** The distribution of distinct anti-PD-L1 clinical response in high- and low-CuRGscore subgroups in the IMvigor210 cohort (chi-square test). PR, Partial Response; PD, Progressive Disease; SD, Stable Disease; and CR, Complete Response.

Since only 20% of patients with cancer benefit from ICB therapy, an accurate alternate predictor is vital (Mamdani et al., 2022). Hence, we first calculated the TIDE score to evaluate the predictive ability of CuRGscore for immunotherapy in LUAD. We found that the patients in the high-CuRGscore group had a high TIDE score (Figure 7A). Also, these patients had higher microsatellite instability (MSI) and cancer-associated fibroblast (CAF) scores (Figures 7B, C), which is suppressive for the function of immune cells. The external testing cohort exhibited similar results (Figures 7D–F). Moreover, Spearman correlation analysis showed a positive correlation between the CuRGscores and the TIDE scores in both training and external testing cohort (Figures 7G, H). These findings indicated that patients in the low-CuRGscore group respond better to ICB therapy than those in the high-CuRGscore group. We further found a negative correlation between the CuRGscores and immunophenoscores in the TCGA-LUAD dataset (Figure 7I). Group-wise comparison also demonstrated a higher immunophenoscore in the low-CuRGscore group (Figure 7J), strengthening the hypothesis that patients of the low-CuRGscore group respond better to ICB immunotherapy. To test the predictive potential of the CuRGscores in the clinical setting, we determined the relationship between the anti-PD-1/PD-L1 immunotherapeutic response and CuRGscore in the GSE78220 dataset with pre-treatment melanomas undergoing anti-PD-1 therapy (Hugo et al., 2016). We found that patients with complete response (CR) and partial response (PR) were mainly fell into low score area as well as patients with progressive disease (PD) were mostly in high score area (Figure 7K). The Chi-square test indicated that 71% of patients in the low-rated CuRGScore group benefited from anti-PD-1 therapy (CR or PR), compared with the high-rated group (36%, Figure 7L). Finally, we employed pre-treatment urothelial cancer samples from a large phase II trial (IMvigor210) with anti-PD-L1 to evaluate the prognosis of patients. Kaplan-Meier survival curve revealed that patients in the low-CuRGscore group exhibited a better OS from the immune therapy than those in the high-CuRGscore group (Figure 7M). Patients with CR/PR showed a significantly lower CuRGscore than those with stable Disease (SD)/PD (Figure 7N). The percentage of patients with CR/PR in the low-CuRGScore group was about 2.5 times higher than that in the high-CuRGscore group (Figure 7O). Collectively, these results indicated that patients in the low-CuRGscore group have a better chance of benefiting from ICB immunotherapy.

### 3.7 CuRGscore directs chemotherapeutics for patients with LUAD

**FIGURE 8:**
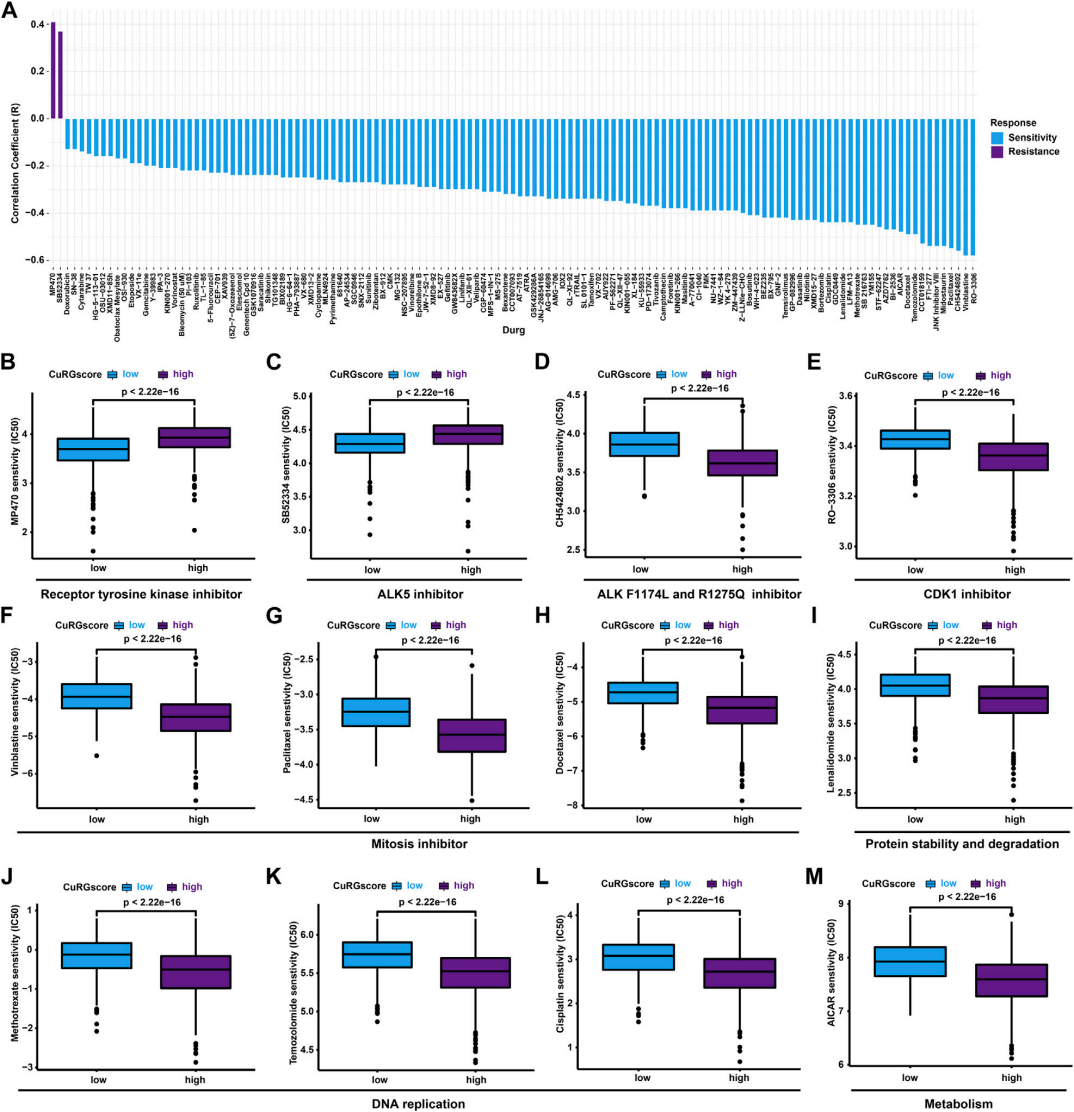
Chemotherapeutic responses in high- and low-CuRGscore patients with LUAD. **(A)** Common drug sensitivity and resistance for high- and low-risk patients with LUAD using pRRophetic analysis in the training and external validation cohorts. **(B, C)** Drug resistance for receptor tyrosine kinases inhibitor, MP470 **(B)**, and ALK5 inhibitor, SB52334 **(C)** in high-risk patients with LUAD. **(D, E)** Drug sensitivity for ALK F1174L and ALK R1275Q inhibitor, CH5424802 **(D)**, and CDK1 inhibitor, RO-3306 **(E)**, in high-risk patients with LUAD. **(F–H)** Chemotherapeutic sensitivity for mitosis inhibitors, such as vinblastine **(F)**, paclitaxel **(G)** and docetaxel **(H)** in high-risk patients with LUAD. **(I)** Chemotherapeutic sensitivity for protein stability and degradation (lenalidomide) in high-risk patients with LUAD. **(J–L)** Chemotherapeutic sensitivity for DNA replication, including vinblastine **(J)**, paclitaxel **(K)**, and Cisplatin **(L)** in high-risk patients with LUAD. **(M)** Drug sensitivity for metabolism regulator, AICAR, in high-risk patients with LUAD.

To evaluate the predictive potential of CuRGscore on drug sensitivity, pRRophetic analysis was performed to estimate the IC50 of chemotherapeutics with a ridge regression model for high- and low-CuRGscore patients. We found 129 drugs coexisted in the training and external testing cohorts, significantly associated with CuRGscores, including 2 resistant and 127 sensitive drugs (Figure 8A and Supplementary Table S7). Notably, two receptor tyrosine kinase inhibitors, MP470 and SB52334 (ALK5 inhibitor), exhibited drug resistance in patients of the high-CuRGscore group (Figures 8B, C). While patients in the high-CuRGscore group were more sensitive to ALK F1174L and ALK R1275Q inhibitor, CH5424802 (Figure 8D), indicating that these patients had more ALK mutation. An ATP-competitive and selective CDK1 inhibitor, RO-3306, had the highest drug sensitivity and Spearman correlation for patients of the high-CuRGscore group among all 127 sensitivity drugs in both the training and testing cohorts (Figure 8E). Vinblastine, paclitaxel, and docetaxel, disturbing the microtubule cytoskeleton in mitosis, showed better chemotherapeutic potential for patients in the high-CuRGscore group (Figures 8F–H). Protein stability and degradation regulator, lenalidomide, as well as three DNA replication inhibitors, vinblastine, paclitaxel, and Cisplatin, also revealed better chemotherapeutic sensitivity in the high-CuRGscore group (Figures 8I–L). Additionally, the metabolism regulator, AICAR, was also more sensitive in the high-CuRGscore group than in the low-CuRGscore group (Figure 8M). Together, our results suggested that CuRGscore could be used for chemotherapeutic evaluation in patients with LUAD.

## 4 Discussion

Although ICB immunotherapy is a milestone for cancer treatment, only a minority of patients with cancer could benefit from it. For example, PD-L1 expresses in at least 50% of tumor cells in patients with advanced NSCLC, only 45.2% of these patients benefited from the anti-PD-1 antibody, pembrolizumab (Garon et al., 2015). The heterogeneity of TEX was considered an essential determinant of the immunotherapeutic response of cancer (Liu et al., 2020; Inflammatory et al., 2021). Hence, developing a virtual microdissection analytical approach to resolve the heterogeneity of TEX is eminent. Herein, based on 1012 LUAD transcriptional data from five datasets, we employed unsupervised hierarchical clustering and PCA algorithm to construct three CuRGclusters, three geneClusters, and CuRGscore. These molecular subtypes could distinguish the phenotypes of TEX and malignant grades of TME to reliably predict prognosis as well as immunotherapeutic and chemotherapeutic responses in patients with LUAD.

Multiple signatures of TEX have been identified from cancers, including NSCLC (Guo et al., 2018). In our study, identified CuRGs-related clusters and CuRGsores could significantly distinguish these TEX signatures. Furthermore, four gene signatures of TEX phenotype, terminal, GZMK+, OXPHOS- and TCF7+ TEX subtypes, were identified from pan-cancer single-cell sequencing data (Zheng et al., 2021). As progenitors of TEX, the TCF7+ phenotype had stem cell-like capacity, and they differentiated into intermediate phenotype with a loss of TCF1. Studies have shown that the terminal phenotype cannot be functionally reinvigorated through ICB therapy (Beltra et al., 2020). Compared with other molecular subtypes, CuRGcluster C, geneCluster B, and high-CuRGscore group with poor prognoses had a higher fraction in terminal TEX, GZMK+ TEX, and OXPHOS- TEX. Especially, the TEX terminal phenotype was remarkably related to the CuRGscore (R = 0.62 and *p* < 0.001). These demonstrated that CuRGscore was a good signature to direct TEX phenotype and predict prognosis in LUAD.

Dysfunction in IFN, TNF, IL2, IL6, and glycolysis signaling pathways, as well as high expression of immunosuppressive receptors such as *PDCD1*, *CTLA4*, *HAVCR2*, *TIGIT*, *LAG3,* and *SIGLEC7*, are classic characteristics of TEX (Blank et al., 2019; Saleh et al., 2020). Our study found that CuRGcluster C, geneCluster B, and high-CuRGscore groups represented TEX characteristics with poor prognosis. TEX is also regulated by a network of transcription factors and multiple inflammatory factors (Collins and Henderson, 2014; Fang et al., 2022). For example, high expression levels of *EOMES* could accelerate the exhaustion of anti-tumor CD8^+^ T cells (Li et al., 2018). We found that *EOMES* was also highly expressed in geneCluster B and high-CuRGscore groups with a short OS. Moreover, inflammatory factors, *L1RN*, *IL10*, *CCL18*, *TGFB1*, *IL1B,* and *CCL2*, were enhanced in CuRGcluster C, geneCluster B, and high-CuRGscore groups compared with the other groups. Interestingly, copper importer *SLC31A1* was significantly and positively associated with TEX phenotypes and checkpoint genes, while copper exporters, ATP7A and ATP7B, were negatively correlated with TEX phenotypes and checkpoint genes. Recently, two pieces of research also demonstrated that *SLC31A1*-dependent copper absorption increases the malignancy grade of pancreatic and breast cancers (Yu et al., 2019; Li et al., 2022). We also observed that three anti-cuproptosis genes (*CDKN2A*, *GLS* and *MTF1*) were positively associated with TEX characteristics. Additionally, seven pro-cuproptosis genes (*DLAT*, *DLD*, *FDX1*, *LIAS*, *LIPT1*, *PDHA1,* and *PDHB*) were also positively associated with TEX characteristics. These results were more significant in the external validation dataset, which reconfirmed the conclusion that cuproptosis was involved in the development of TEX of LUAD.

Besides TEX, other immunosuppressive characteristics are also essential to determine the degree of malignancy of TME, which influences immuno- and chemotherapeutic responses. Studies have shown that Treg, MDSC, and TAM extensively infiltrate advanced NSCLC (Liang et al., 2022). In this study, CuRGcluster C, geneCluster B, and high-CuRGscore subtypes were significantly enriched in infiltrating Treg, MDSC, and TAM. Moreover, CuRGscore was significantly related to the TIDE score and immunophenoscore, as well as can predict immunotherapeutic responses and drug sensitivity. Our study showed that two receptor tyrosine kinase inhibitors, MP470 (c-Kit, PDGFα, Flt3 inhibitor) and SB52334 (ALK5 inhibitor), showed drug resistance in patients with high CuRGscore. Although the inhibitors have not been used in clinic, they are promising antitumor drugs (Mahadevan et al., 2007; Qi et al., 2009). Vinblastine (Velban), paclitaxel (Taxol), docetaxel (Taxotere) and cisplatin (Platinol AQ) as clinical antitumor agents, are more sensitive in high CuRGscore group, which further indicates that CuRGscore can guide the use of drugs. Collectively, these results indicate that cuproptosis is involved in the development of immunosuppressive and malignant TME in patients with LUAD.

## 5 Conclusion

Our study demonstrated the extensive regulatory mechanisms of cuproptosis on TEX. Our results indicate that CuRGs-related molecular subtypes and scores could distinguish the phenotypes of TEX to reliably predict prognosis and direct more effective immunotherapeutic and chemotherapeutic strategies for patients with LUAD.

## Data Availability

Publicly available datasets were analyzed in this study. This data can be found here: https://portal.gdc.cancer.gov, https://www.ncbi.nlm.nih.gov/geo/, http://research-pub.gene.com/IMvigor210Core Biologies/.
