# Supplementary figures and images for: Cuproptosis-related molecular subtypes direct T cell exhaustion phenotypes and therapeutic strategies for patients with lung adenocarcinoma
Source: Front Pharmacol. 2023 Apr 11;14:1146468. doi: 10.3389/fphar.2023.1146468 (PMC10126426; doi:10.3389/fphar.2023.1146468)

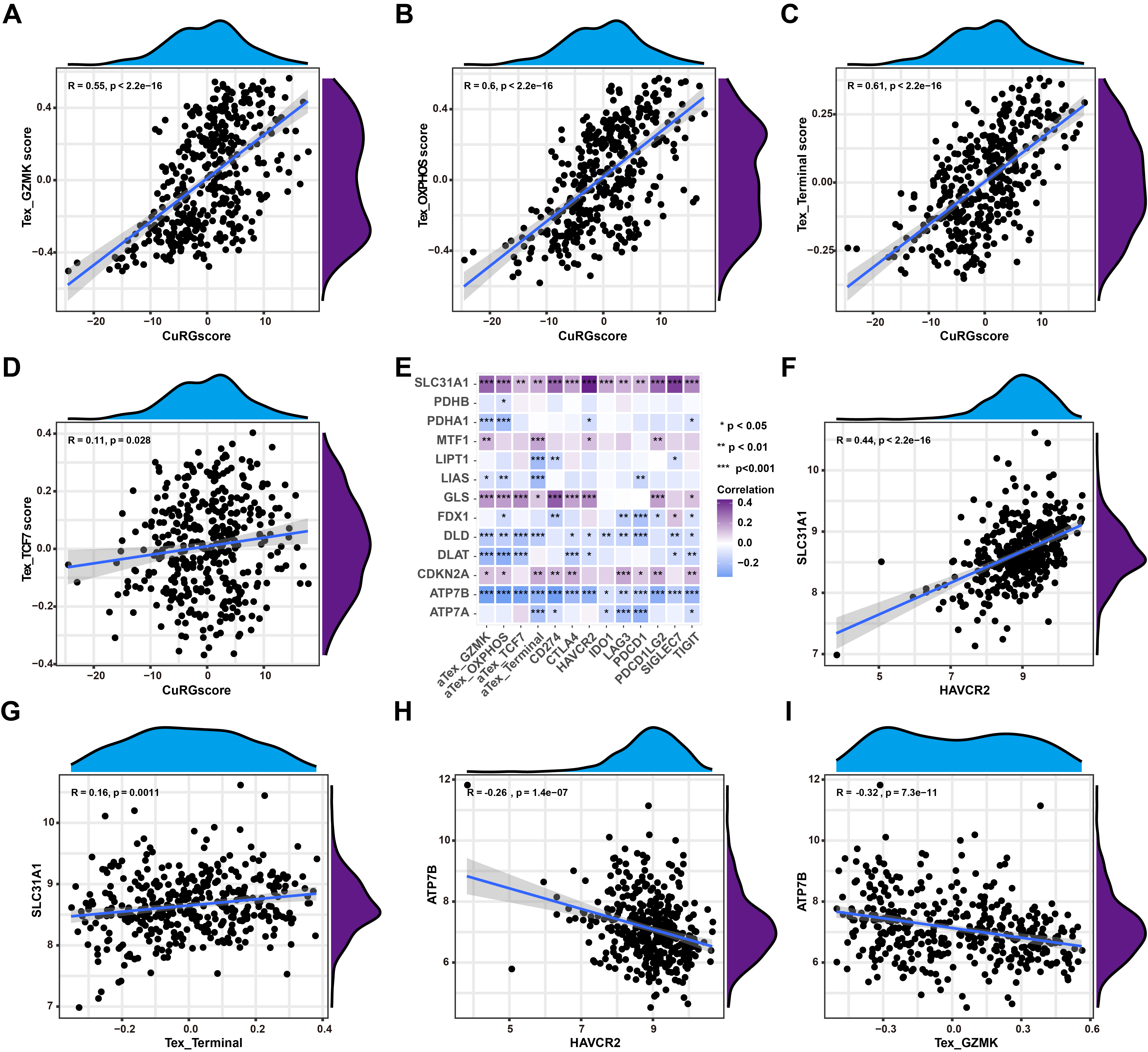

Supplement: Supplementary file 3 [file Image3.jpg]

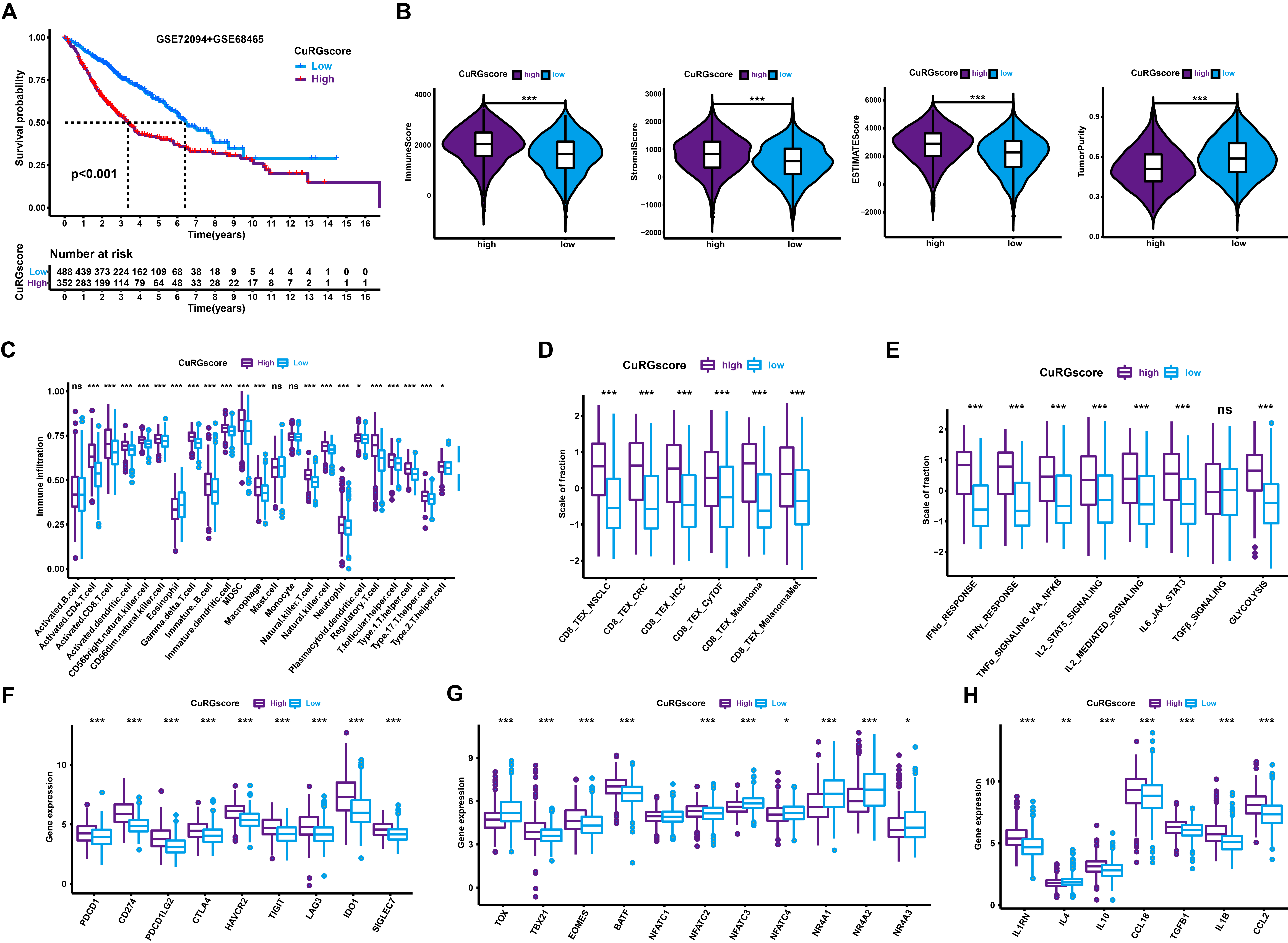

Supplement: Supplementary file 4 [file Image2.jpg]

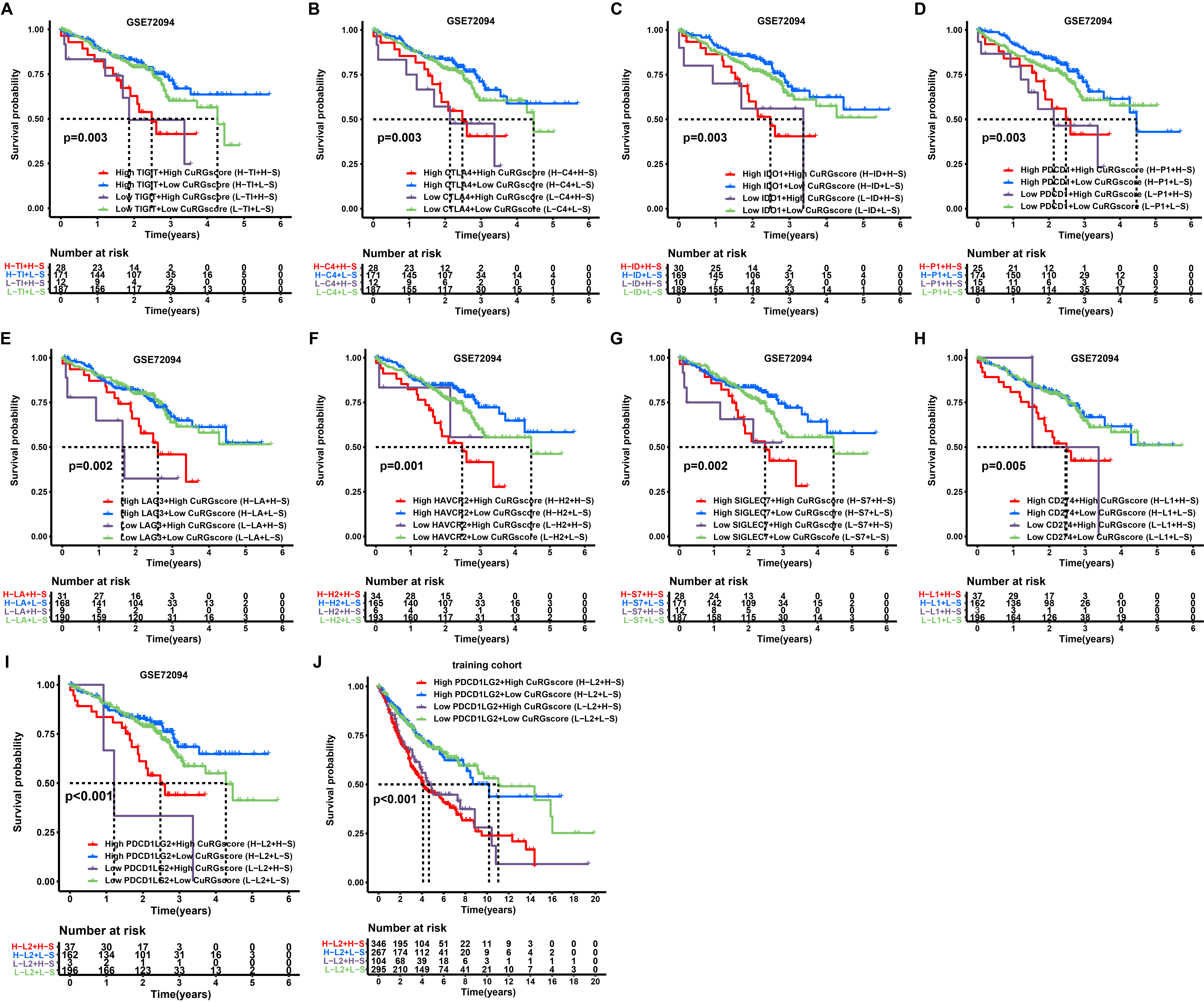

Supplement: Supplementary file 8 [file Image4.jpg]

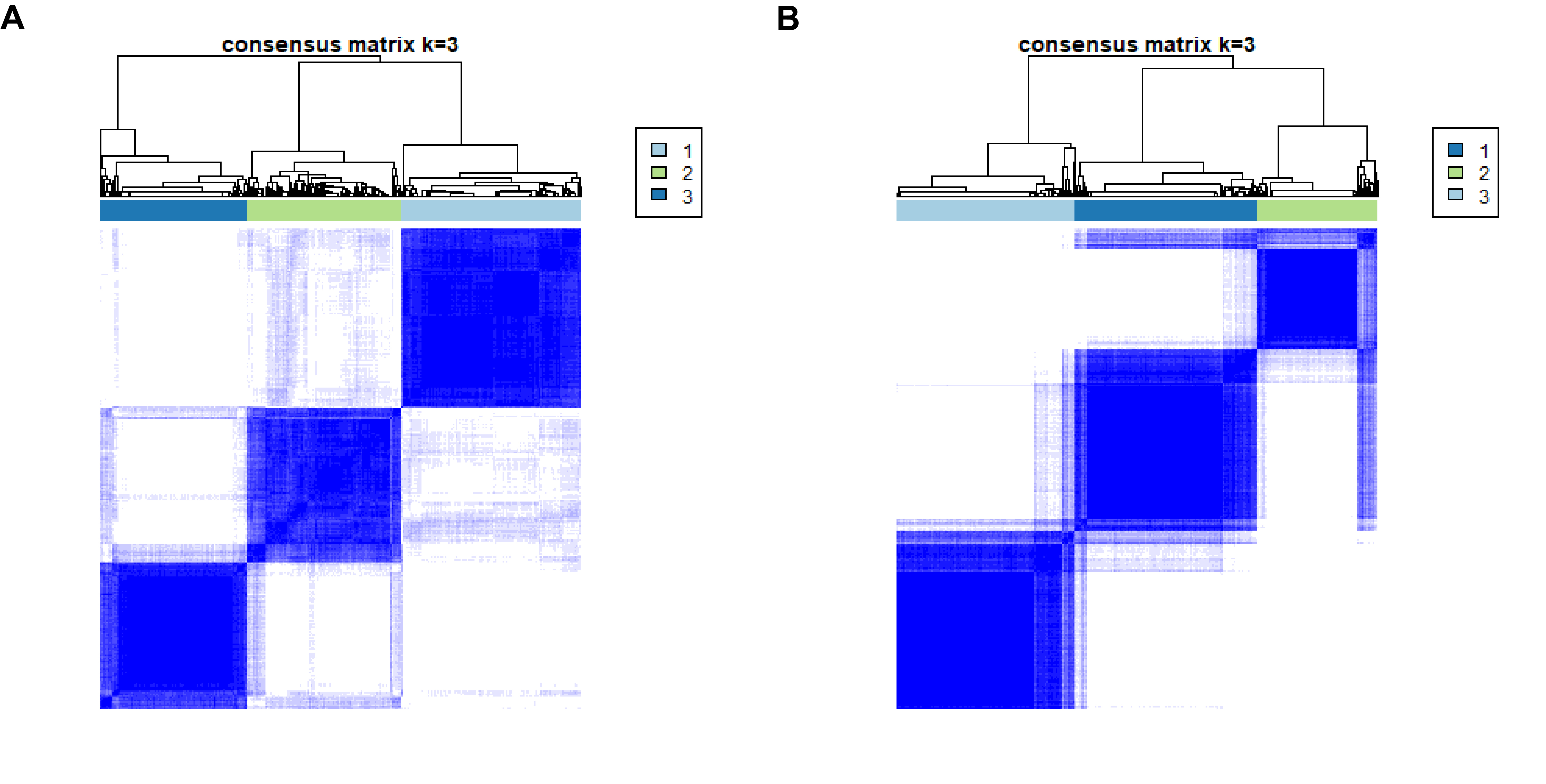

Supplement: Supplementary file 11 [file Image1.jpg]
